# Usefulness of Interleukin-18 as a Diagnostic Biomarker to Differentiate Adult-Onset Still’s Disease With/Without Macrophage Activation Syndrome From Other Secondary Hemophagocytic Lymphohistiocytosis in Adults

**DOI:** 10.3389/fimmu.2021.750114

**Published:** 2021-10-08

**Authors:** Toshihiko Shiga, Yuji Nozaki, Daisuke Tomita, Kazuya Kishimoto, Yasuaki Hirooka, Koji Kinoshita, Masanori Funauchi, Itaru Matsumura

**Affiliations:** ^1^Department of Hematology and Rheumatology, Kindai University School of Medicine, Osaka, Japan; ^2^Department of Rheumatology, Kindai University Nara Hospital, Nara, Japan

**Keywords:** interleukin-18, adult-onset Still’s disease, hemophagocytic lymphohistiocytosis, macrophage activation syndrome, diagnostic biomarker

## Abstract

**Background:**

Interleukin (IL)-18 is markedly elevated in systemic inflammatory diseases that cause the ‘cytokine storm’ such as adult-onset Still’s disease (AOSD) and hemophagocytic lymphohistiocytosis (HLH). The differences in IL-18 between AOSD and HLH, especially in adults, is uncertain. Macrophage activation syndrome (MAS), a form of secondary HLH, is often difficult to differentiate cases of AOSD that include MAS from other secondary HLH. In this case-control study, we investigated whether serum IL-18 levels could be a useful biomarker for the differential diagnosis of AOSD with or without MAS (AOSD group) and other secondary HLH in adults (adult HLH group).

**Patients and Methods:**

We enrolled 46 patients diagnosed with AOSD including 9 patients with MAS and 31 patients in the adult HLH group, which excluded AOSD-associated MAS. The clinical features and laboratory data were compared between the AOSD and adult HLH groups. In addition, we subdivided the AOSD group (with or without MAS) and the adult HLH group (whether lymphoma-associated or not) and compared the four groups. A logistic regression analysis was used to identify factors with high efficacy in differentiating the two groups, followed by a receiver operating characteristic (ROC) curve analysis to evaluate the differential diagnostic ability of IL-18. We analyzed the correlation between IL-18 and various laboratory parameters in the AOSD group.

**Results:**

Serum IL-18 levels of patients in the AOSD groups were significantly higher than those of the adult HLH groups, and were closely correlated with ferritin, soluble interleukin-2 receptor (sIL-2R), and other laboratory data. Univariate and multivariate logistic regression analyses revealed that IL-18, sIL-2R, and ‘arthralgia or arthritis’ are independent factors useful in the differential diagnosis of AOSD from adult HLH. In the differential diagnosis of both groups, the area under the curve obtained from the ROC curve of IL-18 with a cutoff value of 18,550 pg/mL was 0.91 (95% confidence interval 0.83–1.00; sensitivity 90.3%, specificity 93.5%), and the differential diagnosis ability of IL-18 was superior to that of other laboratory data.

**Conclusions:**

IL-18 could be a useful biomarker for the differential diagnosis of AOSD and adult HLH.

## Introduction

Adult-onset Still’s disease (AOSD) is a rare, polygenic, systemic autoinflammatory disease characterized by recurrent fever, skin rash (typically salmon-pink in color), arthralgia or arthritis, sore throat, leukocytosis with neutrophilia, lymphadenopathy, splenomegaly, and liver dysfunction (1, 2). The etiology of AOSD is unknown, but the following are recognized as central to the pathogenesis of AOSD: an abnormal activation of innate immune cells (e.g., neutrophils, monocytes, and macrophages) and the overproduction of cytokines such as interleukin (IL)-1β, IL-6, tumor necrosis factor-alpha (TNF-α), and IL-18 (1–4). The diagnosis of AOSD is based on a combination of clinical and laboratory findings, excluding infections (especially sepsis and viral infections), malignant neoplasms (especially lymphoma), and inflammatory diseases (especially polyarthritis nodosa) (5, 6). The serum level of ferritin is frequently used as a practical tool for the diagnosis of AOSD, but its diagnostic specificity is low and of limited value in the diagnosis of AOSD (3, 5, 7). The diagnosis of AOSD is thus often difficult, and we have sought to identify specific diagnostic markers for AOSD.

Serum levels of various cytokines and chemokines have been reported to be useful in the diagnosis of AOSD (8), and IL-18 has been found to be markedly elevated in the serum of AOSD patients in several studies (1, 9). IL-18 has been reported to be associated with the disease activity of AOSD (10, 11), and it may be useful as an indicator of therapeutic efficacy (12). The question of whether IL-18 could be a target for the treatment of AOSD is being investigated (13). IL-18 may play a particularly important role in the pathophysiology of AOSD, but whether IL-18 may be useful as its diagnostic biomarker remains to be determined.

Hemophagocytic lymphohistiocytosis (HLH) and hemophagocytic syndrome (HPS) are life-threatening inflammatory systemic disorders, and their clinical features (e.g., fever and splenomegaly) and laboratory data (e.g., elevated transaminases and hyperferritinemia) are very similar to those of AOSD (14). HLH in adults is usually classified as secondary (reactive), which is subdivided into infectious, neoplastic, and autoimmune-related secondary HLH (14, 15).

Macrophage activation syndrome (MAS), an acquired form of HLH that occurs in autoimmune diseases, is a common and serious complication of AOSD, and it is especially difficult to distinguish AOSD-related MAS from HLH or MAS with other underlying diseases (16). It was recently revealed that serum IL-18 levels are increased in primary (genetic) and secondary HLH, especially in pediatric patients, and several reports have compared the serum IL-18 levels between pediatric HLH cases and patients with systemic juvenile idiopathic arthritis (sJIA), a homolog of AOSD in children (17). There have been few similar comparative studies between AOSD and adult HLH. We thus conducted the present study to investigate whether serum IL-18 levels could be a useful biomarker for the differential diagnosis of AOSD with or without MAS and other forms of secondary HLH in adults.

## Methods

### Patients: the AOSD and Adult HLH Groups

This was an observational case-control study, and all medical records were analyzed retrospectively. All patients in this study between January 2012 and December 2020 were diagnosed by several specialists in Rheumatology and Hematology. We analyzed the cases that could be enrolled in our study from the names of diseases listed in the medical records. We defined the AOSD group as AOSD patients with or without MAS. In this study, MAS was defined as HLH related to AOSD. We defined the adult HLH group as patients with all secondary HLH except MAS (associated with AOSD), and the HLH patients related to other rheumatic diseases was assigned to the adult HLH group. All patients in the two groups developed the disease in adulthood, >20 years old. The 46 patients with AOSD group and the 31 patients with adult HLH diagnosed at the Kindai University School of Medicine in Osaka were enrolled in the study. The AOSD patients met the Yamaguchi criteria after the exclusion of infections, tumors, and other autoimmune diseases (5). In this study, it was essential to prove hemophagocytosis in bone marrow for the diagnosis of HLH and MAS. In addition, the diagnosis was based on meeting 5 or more of 7 the HLH-2004 non-molecular/non-genetic criteria (including hemophagocytosis in bone tissue and excluding low or absent natural killer　[NK]- cell activity) (18). HLH is usually classified according to the etiology (14), but in the present study we divided the HLH cases into lymphoma-associated hemophagocytic syndrome (LAHS) and non-LAHS. The background of non-LAHS included autoimmune diseases in six patients (systemic lupus erythematosus [SLE]: n=5, dermatomyositis: n=1), infectious diseases in three patients (sepsis: n=2, multiple muscle abscesses: n=1), and others in five patients (breast cancer with systemic metastasis: n=1, after hematopoietic stem cell transplantation: n=1, unknown: n=3). Clinically active patients with AOSD and adult HLH patients were included in this study. The disease activity of the AOSD patients without MAS was assessed with a modified Pouchot score, and we defined the cases with scores higher than four as having clinically active disease (19, 20). We also considered all of the AOSD with MAS and adult HLH patients enrolled in this study as clinically active.

### Blood Samples

In cases of suspected AOSD and cases of febrile illnesses that were difficult to distinguish from AOSD, we have measured serum IL-18 and IL-6 levels after obtaining patient’s consent to measure for diagnostic purposes since January 2012. In the present study, blood samples were collected from all patients at their diagnosis of AOSD or adult HLH with or without therapeutic interventions involving steroids, and the serum IL-18 and IL-6 levels were measured together with other routine blood tests as summarized in **Table 1**.

**Table 1:** The demographic, clinical and laboratory characteristics of the AOSD and adult HLH group.

Characteristics	AOSD (N = 46)	Adult HLH (N = 31)	p-value
Sex: female, n (%)	36 (78.3)	13 (41.9)	0.0017
Age, years [IQR]	58.5 [40–70]	67.0 [49–75]	0.053
Clinical features:			
Fever >39°C, n (%)	45 (97.8)	29 (94.4)	0.58
Skin rash, n (%)	42 (91.3)	17 (45.2)	<0.0001
Arthralgia/arthritis, n (%)	35 (76.1)	9 (29.0)	<0.0001
Myalgia	11 (23.9)	4 (12.9)	0.26
Sore throat, n (%)	31 (67.4)	6 (19.4)	<0.0001
Lymphadenopathy, n (%)	31 (67.4)	19 (61.3)	0.63
Splenomegaly, n (%)	25 (54.4)	17 (54.8)	>0.99
Liver dysfunction, n (%)	36 (78.3)	28 (90.3)	0.22
Serositis, n (%)	12 (26.1)	10 (32.3)	0.61
DIC, n (%)	9 (19.6)	12 (38.7)	0.074
MAS, n (%)	9 (19.6)	–	–
Laboratory data:			
IL-18, pg/mL [IQR]	91,900 [47,250–15,2750]	2,070 [1,380–7,400]	<0.0001
IL-6, pg/mL [IQR]	46.7 [30.2–89.3]	71.9 [29.8–123.0]	0.41
Ferritin, ng/mL [IQR]	5,651 [2,294–22,879]	2,666 [1,124–7,365]	0.062
sIL-2R, U/mL [IQR]	1,517 [945–2,148]	4,860 [2,033–12,813]	<0.0001
CRP, mg/mL [IQR]	8.4 [4.2–15.6]	7.7 [1.3–11.7]	0.19
LDH, IU/L [IQR]	494 [404–895]	704 [421–1,167]	0.13
AST, IU/L [IQR]	61 [28–112]	97 [49–201]	0.061
ALT, IU/L [IQR]	39 [15–77]	61 [31–125]	0.059
Trig, mg/dL [IQR]	150 [103-181], N’=41	171 [137-242], N’=27	0.031
Fib, mg/dL [IQR]	430 [308-563], N’=43	308 [223-424], N’=30	0.0023
HScore [IQR]	146 [119-170], N’=40	189 [160-231], N’=27	<0.0001
ANC,/μL [IQR]	8417 [5469-11877]	3080 [1331-6504]	<0.0001
ALC,/μL [IQR]	910 [668-1281]	570 [288-1357]	0.071
RF-positive, n (%)	4 (9.1), N’=44	7 (26.9), N=26’	0.086
ANA-positive, n (%)	7 (15.6), N’=45	8 (28.6), N=28’	0.24
Treatment before diagnosis, n (%)	8 (17.4)	8 (25.8)	0.4
Steroid (prednisolone), n (%)	8 (17.4)	7 (22.6)	0.57
Prednisolone dose, mg/day [IQR]	25.0 [11.9– 40.0]	15.0 [10–30]	0.55
Immunosuppressant, n (%)	4 (8.7)	1 (3.2)	–
Biologic DMARD, n (%)	–	1 (3.2)	–

Data are numbers (with percentage) or median [interquartile range]. N, total number of patients in each group; N’, number of patients with missing the data; n, number of patients who met each endpoint. ALC, absolute lymphocyte count; ALT, alanine transaminase; ANA, anti-nuclear antibody; ANC, absolute neutrophil count; AST, aspartrate transaminase; CRP, C-reactive protein; DIC, disseminated intravascular coagulation; DMARD, disease-modifying antirheumatic drug; Fib; fibrinogen; IL, interleukin; LDH, L-lactate dehydrogenase; MAS, macrophage activation syndrome; RF, rheumatoid factor; sIL-2R, soluble interleukin 2 receptor; Trig, triglyceride.

This study complied with the standards of the Declaration of Helsinki and the Ethical Guidelines for Medical and Health Research Involving Human Subjects (Ministry of Education, Culture, Sports, Science and Technology, and Ministry of Health, Labor and Welfare of Japan), and was approved by the Research Ethics Committee of the Faculty of Medicine of Kindai University (approval no. R02-218). Witten informed consent from each patient for participation in the study is not necessarily required, because this study used blood test results collected for the purpose of diagnosis in daily practice, and the data were retrospective and non-invasive in nature and analyzed anonymously, as stipulated in the above ethical guideline in Japan. However, we describe the details of this study on our website (https://www.med.kindai.ac.jp/naika3/index.html), which is accessible to all patients, and give them the right to refuse to participate in clinical research. Furthermore, we have obtained written informed consent as much as possible to enroll in this study for cases who are continuing to visit our hospital.

### Measurement of Serum IL-18, IL-6 and sIL-2R Levels

The serum concentrations of IL-18 were assessed using a sandwich enzyme-linked immunosorbent assay (ELISA) according to the manufacturer’s instructions (Medical & Biological Laboratories [MBL], Nagoya, Japan; reference range, unknown). There has been no study that clearly states the reference value in the report on AOSD using MBL’s kit to measure serum IL-18. However, according to the attached material of the Human IL-18 ELISA kit of MBL, serum samples for 46 healthy blood donors were assayed by the measurement kit, and the concentrations of human IL-18 in normal sera were mean ± SD; 126.0 ± 44.5 pg/mL, maximum; 257.8 pg/mL, and minimum; 36.1 pg/mL. All serum IL-18 samples were measured up to 5000 pg/mL with a 5-fold dilution at the initial dilution. For samples of 5000 pg/mL or more, the data was measured by further diluting 50 times, and finally, it was confirmed that the data was within the measurement range by diluting 250 times. The serum concentrations of IL-6 were measured using a chemiluminescent enzyme immunoassay (CLEIA) according to the manufacturer’s instructions (Fujirebio, Tokyo, Japan; reference range, <4.0pg/mL). Serum IL-6 was first measured in undiluted solution, and if the value exceeds 1000 pg/mL, the test was repeated after dilution 100 times. Serum sIL-2R levels were measured by a CLEIA according to the manufacturer’s instructions (LSI Medience, Tokyo, Japan; reference range, 121- 613 U/mL). In the case of serum sIL-2R, it was also measured in undiluted solution, and if the value exceeds 100,000 U/mL, it was diluted 10 times and retested.

### Statistical Analyses

Continuous variables are expressed as the median (interquartile range [IQR]), and categorical variables are expressed as the number (n) and percentage (%). The Mann-Whitney U-test or Fisher’s exact test were used to compared demographic characteristics, clinical features and laboratory findings between the AOSD and adult HLH groups. Data among four groups were analyzed using the Kruskal-Wallis test followed by *post hoc* Dunn’s test with Bonferroni correction. A logistic regression analysis was performed to identify the variables that could be used to differentiate AOSD from adult HLH. The odds ratios (ORs) were calculated by adjusting 10,000 pg/mL to 1 unit for IL-18 and by adjusting 1.000 U/mL to 1 unit for sIL-2R and ferritin. We subsequently performed a receiver operating characteristic (ROC) curve analysis for IL-18 distinguishing the AOSD group from the adult HLH group, and we calculated the area under the curve (AUC), cutoff value, sensitivity, and specificity. Spearman’s rank correlation test was used to test the association between serum IL-18 and other laboratory data in the AOSD group. P-values<0.05 were considered significant. The statistical analyses were performed using SPSS for windows ver. 22.0 (IBM Japan, Tokyo, Japan) and GraphPad Prism 9 software (GraphPad Software, San Diego, CA).

## Results

### Clinical and Laboratory Characteristics of the AOSD and Adult HLH Groups

**Table 1** summarizes the demographic characteristics, clinical features, and laboratory data of the 46 patients in the AOSD group and the 31 patients in the adult HLH group at the time of blood sampling. For triglyceride (Trig), fibrinogen (Fib), HScore, rheumatoid factor and ant-nuclear antibody, there were several missing data in each group, as shown in **Tables 1**, **2**. The AOSD group had a significantly higher number and proportion of females (p=0.0017) and tended to be younger (p=0.053). The frequencies of skin rash, arthralgia or arthritis, and sore throat were each significantly higher in the AOSD group (p<0.0001). The AOSD group had significantly higher serum IL-18 levels (p<0.0001), absolute neutrophil count (ANC) (p<0.0001) and Fib (p=0.0023), and lower sIL-2R levels (p<0.0001), HScore (p< 0.0001) and Trig (p=0.031) compared to the adult HLH group. Ferritin tended to be higher in the AOSD group but was not significantly different between the two groups (p=0.062). IL-6 was not significantly different between the two groups.

**Table 2:** The laboratory characteristics of the AOSD MAS-, AOSD MAS+, LAHS, and non-LAHS group.

	① AOSD MAS - (N=37)	② AOSD MAS + (N=9)	③ LAHS (N=17)	④ non-LAHS (N=14)	p-value	p-value (Bonferroni correction)					
					for all	① vs. ②	① vs. ③	① vs. ④	② vs. ③	② vs. ④	③ vs. ④
IL-18	79,500 [46,150-135,500]	152,000 [81,200-206,500]	6,450 [1,890-7,675]	1,740 [866-18,442]	<0.0001	>0.99	<0.0001	0.00013	0.00010	0.00023	>0.99
IL-6	41.8 [30.5-70.5]	89.2 [32.5-214.0]	73.9 [32.7-142.5]	68.4 [22.8-114.3]	0.33	–	–	–	–	–	–
Ferritin	4,912 [1,729-17,138]	35,328 [6,527-64,774]	2,498 [645-6,002]	3,706 [1,741-8,386]	0.0050	0.020	>0.99	>0.99	0.0026	0.045	>0.99
sIL2R	1,473 [942-2,033]	2,084 [1,430-5,934]	9,468 [4,200-16,112]	2,395 [1,610-5,767]	<0.0001	0.34	<0.0001	0.068	0.28	>0.99	0.25
CRP	7.8 [4.6-14.2]	12.2 [2.1-20.4]	9.0 [4.1-12.4]	4.5 [0.4-10.9]	0.14	–	–	–	–	–	–
LDH	464 [360-627]	834 [397-1,084]	481 [367-1,180]	711 [563-1,080]	0.18	–	–	–	–	–	–
AST	54 [28-85]	93 [45-205]	60 [46-136]	154 [52-475]	0.049	0.85	>0.99	0.047	>0.99	>0.99	0.84
ALT	34 [13-68]	61 [27-246]	45 [31-117]	68 [32-213]	0.12	–	–	–	–	–	–
ANC	8,379 [5,776-10,962]	10,193 [3,823-18,012]	3,192 [1,286-6,426]	2,696 [1,576-6,507]	<0.0001	>0.99	0.0022	0.0026	0.035	0.031	>0.99
ALC	964 [715-1,288]	623 [381-945]	932 [310-1,797]	476 [277-955]	0.017	0.36	>0.99	0.020	>0.99	>0.99	0.39
Trig	150 [97-175], N’=33	158 [115-209], N’=8	207 [132-246], N’=15	170 [140-185], N’=12	0.13	–	–	–	–	–	–
Fib	497 [359-587], N’=34	274 [251-433], N’=9	391 [195-453], N’=17	275 [230-381], N’=13	0.00065	0.057	0.063	0.0025	>0.99	>0.99	>0.99
HScore	131 [110-162], N’=32	195 [145-240], N’=8	185 [160-235], N’=15	206 [161-223], N’=12	<0.0001	0.012	0.00059	0.0014	>0.99	>0.99	>0.99

The units for each of data are the same as for **Table 1**. Data are median [interquartile range]. N, total number of patients in each group; N’, number of patients with missing the data; ANC, absolute neutrophil count; ALC, absolute lymphocyte count; ALT, alanine transaminase; AST, aspartrate transaminase; CRP, C-reactive protein; IL, interleukin; LDH, L-lactate dehydrogenase; MAS, macrophage activation syndrome; sIL-2R, soluble interleukin 2 receptor; Trig, triglyceride; Fib, fibrinogen.

Nine of the 46 (19.6%) patients with AOSD presented with MAS. Eight (17.4%) of the AOSD patients and eight (25.8%) of the adult HLH patients had been treated with a steroid (prednisolone) and/or an immunosuppressive agent (methotrexate: n=2, cyclosporin: n=2, cyclophosphamide: n=1) and/or a biologic disease-modifying antirheumatic drug (abatacept: n=1) by the time of diagnosis. However, there was no significant between-group difference regarding the numbers or proportions of patients who received treatment before their diagnosis or in the prednisolone dose.

The details of the 9 patients in the AOSD MAS+ group who met 5 or more of 7 the HLH 2004 criteria are given in **Supplementary Table S1**. The underlying diseases and serum IL-18, sIL-2R and ferritin levels of individual patients in the non-LAHS group are shown in **Supplementary Table S2**.

### Comparisons of Laboratory Characteristics Between the Patients With AOSD Without or With MAS, LAHS, and Non-LAHS Groups

**Figure 1:**
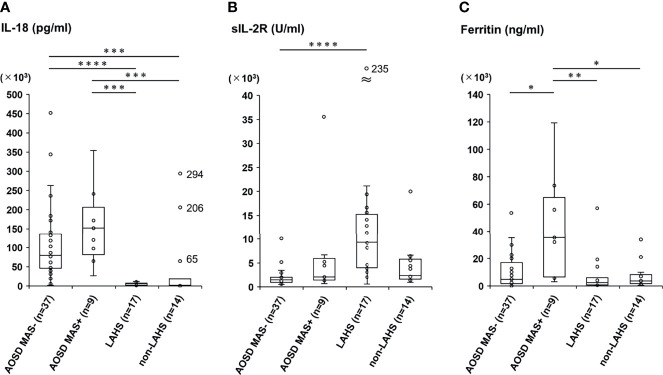
Comparisons of serum levels of IL-18, sIL-2R, and ferritin between the AOSD and adult HLH groups with different subtypes. Serum levels of IL-18 **(A)**, sIL-2R **(B)**, and ferritin **(C)** were compared among patients with AOSD without MAS (AOSD MAS−), those with AOSD with MAS (AOSD MAS+), patients with LAHS, and a non-LAHS group. N corresponds to the number of samples assayed in each group. The Kruskal-Wallis test followed by *post hoc* Dunn’s test with Bonferroni correction was used to compare the four groups; *p<0.05, **p < 0.01, ***p < 0.001, ****p < 0.0001. MAS, macrophage activation syndrome; LAHS, lymphomas-related hemophagocytic syndrome. In panel **(A)**, very high serum IL-18 levels in three patients of the non-LAHS group are indicated by a real number beside the dot (unknown; 294,000 pg/mL, breast cancer with systemic metastasis; 206,000 pg/mL, multiple muscle abscesses; 648,56 pg/mL). In panel **(B)**, a remarkably elevated serum sIL-2R level (235,320 U/mL) in one patient of the LAHS group is indicated by a real number beside the dot.

We divided the AOSD group into the AOSD patients without MAS (the AOSD MAS− group) and those with MAS (the AOSD MAS+ group), and we divided the adult HLH group into the LAHS and non-LAHS subgroups. We then compared only laboratory data among the four groups (**Table 2** and **Figure 1**).

The serum IL-18 levels in the AOSD MAS− group were significantly higher than those in the LAHS group (p<0.0001) and the non-LAHS group (p=0.00013), and similarly, the serum IL-18 levels in the AOSD MAS+ group were significantly higher than those in each subgroup of the adult HLH group (*vs.* LAHS, p=0.00010; *vs.* non-LAHS, p=0.00023). There was no significant difference between the AOSD- and AOSD+ groups regarding IL-18 (**Figure 1A**). The serum levels of sIL-2R in the AOSD MAS− group were significantly lower than those of the LAHS (p<0.0001) (**Figure 1B**). The serum ferritin levels of the AOSD MAS+ group were significantly higher than those in the AOSD MAS− group (p=0.020), LAHS subgroup (p=0.0026), and non-LAHS subgroup (p=0.045) (**Figure 1C**).

ANC was significantly higher in the AOSD MAS+ group compared with the two HLH group. AST, Fib and absolute lymphocyte count (ALC) were significantly different between the AOSD MAS- and non-LAHS groups. HScore in the AOSD MAS- group was significantly lower than in the other three groups.

### Identification of Independent Predictors That Can Be Used for Differentiating the AOSD and adult HLH Groups

We performed a logistics regression analysis to evaluate potential variables that could be used for the differential diagnosis of the AOSD from adult HLH groups (**Table 3**). In the multivariate analysis, arthralgia or arthritis, IL-18, and sIL-2R were extracted as independent predictive indicators. In particular, IL-18 and sIL-2R were more useful biomarkers to differentiate AOSD and adult HLH compared to ferritin (IL-18: OR 1.23, 95%CI 1.09–1.39, p=0.0010; sIL-2R: OR 0.80, 95%CI 0.70–0.92, p=0.0020).

**Table 3:** Univariate and multivariate analyses to differentiate the AOSD and adult HLH groups.

Variable	Univariate analysis		Multivariate analysis	
	OR (95%CI)	p-value	OR (95%CI)	p-value
Sex (female)	4.98 (1.83–13.55)	0.0020		
Age, yrs	0.97 (0.95–1.00)	0.065		
Treatment before diagnosis	1.65 (0.55–5.01)	0.38		
Skin rash	12.45 (3.58–43.30)	<0.0001		
Arthralgia or arthritis	7.78 (2.78–21.78)	<0.0001	5.32 (1.35–20.99)	0.017
Sore throat	8.61 (2.91–25.45)	<0.0001		
IL-18×10^−4^, pg/mL	1.27 (1.12–1.45)	<0.0001	1.23 (1.09–1.39)	0.0010
sIL-2R×10^−3^, U/mL	0.85 (0.75–0.96)	0.0007	0.80 (0.70–0.92)	0.0020
Ferritin×10^−3^, ng/mL	1.04 (1.00–1.07)	0.073		

Age, sex, and treatment before diagnosis were adjusted in the multivariate analysis. The adjusted odds ratio (OR) was calculated by multiplying the estimated regression coefficient by 10,000 for IL-18 and 1,000 for sIL-2R and ferritin. OR, odds ratio; 95%CI, 95%confidence interval; IL, interleukin; sIL-2R, soluble interleukin 2 receptor.

**Figure 2:**
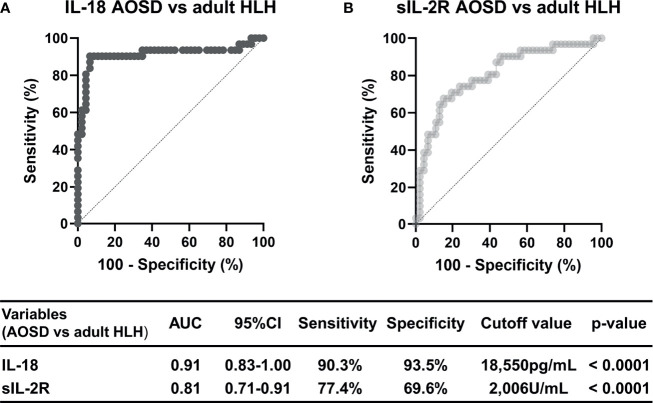
The ROC curve analysis for the differential diagnosis of AOSD and adult HLH based on the serum levels of IL-18 and sIL-2R. The predictive performance of the serum levels of **(A)** IL-18 and **(B)** sIL-2R was validated by the ROC analysis, and the accuracy of differential diagnosis is indicated by the AUC and 95%CI.

An ROC curve analysis was then performed to discriminate the AOSD and adult HLH groups with reference to IL-18 and sIL-2R (**Figure 2**). The results showed that the ROC-AUC with reference to IL-18 was 0.913 (95%CI: 0.83-1.00, p<0.0001). We observed that a serum IL-18 level >18550 pg/mL distinguished the AOSD group from the adult HLH group with 90.3% sensitivity and 93.5% specificity (**Figure 2A**). Using the cutoff value of <2006 U/mL of sIL-2R distinguished the AOSD group from the adult HLH group with 77.4% sensitivity and 69.6% specificity (ROC-AUC 0.81, 95%CI: 0.71–0.91, p<0.0001, **Figure 2B**), indicating that the differential diagnostic ability of IL-18 was superior to that of sIL-2R. The ROC curve analyses regarding IL-18 and sIL-2R for the differential diagnosis between the AOSD patients (n=38) and adult HLH patients (n=23) who were without therapeutic interventions are shown in **Supplementary Figure S1**. In the untreated cases, the ROC-AUC for IL-18 with a cutoff value of 18,550 pg/mL was 0.89 (95%CI: 0.78–0.99; 87.0% sensitivity, 92.1% specificity), and the ROC-AUC of sIL-2R with a cutoff value of 2,122 U/ml was 0.87 (95%CI: 0.77–0.96, sensitivity 82.6%, specificity 79.0%).

### Correlations Between Serum IL-18 Levels and Other Laboratory Data in the AOSD Group

**Figure 3:**
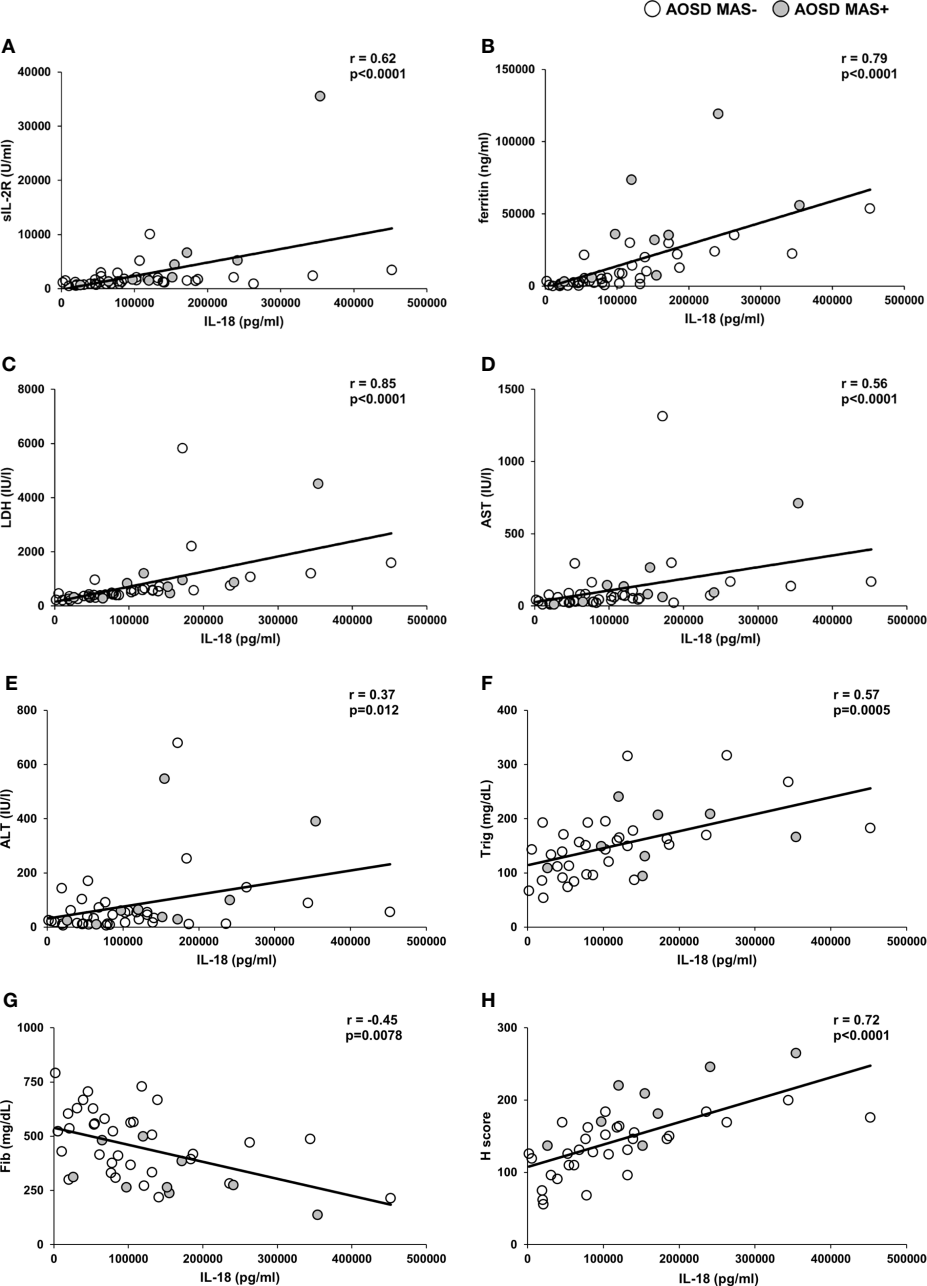
Correlation of serum IL-18 levels and other laboratory data in the AOSD group. Serum IL-18 levels were positively correlated with sIL-2R **(A)**, ferritin **(B)**, LDH **(C)**, AST **(D)**, ALT **(E)**, Trig **(F)** and HScore **(H)**, and were negatively correlated with Fib **(G)**. The correlations were evaluated with Spearman’s rank correlation test. Each dot represents an individual patient; white dots indicate AOSD MAS- patients and grey dots indicate AOSD MAS+ patients. ALT, alanine transaminase; AST, aspartrate transaminase; Fib, fibrinogen; LDH, L-lactate dehydrogenase; Trig, triglyceride.

We also analyzed the correlations between IL-18 and other laboratory data in the AOSD patients (**Figure 3**). The IL-18 levels in serum were strongly correlated with sIL-2R (r=0.62, p<0.0001, **Figure 3A**), ferritin (r=0.79, p<0.0001, **Figure 3B**), LDH (r=0.85, p<0.0001, **Figure 3C**), AST (r=0.56, p<0.0001, **Figure 3D**), Trig (r=0.57, p=0.0005, **Figure 3F**) and HScore (r=0.72, p<0.0001, **Figure 3H**), and weakly associated with ALT (r=0.37, p=0.012, **Figure 3E**). In addition, IL-18 and Fib showed a negative correlation (r=-0.45, p=0.0078, **Figure 3G**). In contrast, IL-18 was not correlated with IL-6, C-reactive protein, ANC and ALC (data not shown).

## Discussion

The results of our analyses demonstrated that serum IL-18 levels could be used as a biomarker with high accuracy for the differential diagnosis of AOSD and adult HLH. AOSD, HLH, and MAS share some similar aspects of their pathogeneses (e.g., an abnormal activation of macrophages and an excessive production of various cytokines), and it was reported that serum IL-18 levels are markedly elevated in each of these diseases (1–3, 16, 21). However, few studies have compared serum IL-18 levels between AOSD, HLH, and MAS in adults (unlike sJIA, HLH and MAS in pediatric patients), because adult HLH and MAS have a lower incidence compared to those in pediatric patients, and few studies of adult HLH or MAS have been performed (14). The effectiveness of the use of serum IL-18 levels to distinguish these diseases has thus not been fully validated. To the best of our knowledge, our present investigation is the only case-control study to directly compare serum IL-18 levels between AOSD with or without MAS and other secondary HLH or MAS in adults, unlike the research regarding sJIA and pediatric HLH or MAS.

To assess the diagnostic ability of IL-18 in AOSD, it is necessary to conduct comparative studies with control diseases that have pathologies and clinical findings similar to those of AOSD. Between 2001, when Kawashima et al. were the first to report that serum IL-18 was markedly elevated in patients with AOSD, and the present investigation’s writing, several studies have compared serum IL-18 levels in AOSD and other inflammatory diseases in adults (8, 10, 12, 22–32). The most common control group in those reports was rheumatic diseases, mainly such as rheumatoid arthritis, SLE, and Sjogren’s syndrome. Serum IL-18 levels in AOSD patients were statistically higher than those in any of the rheumatic diseases in those studies. The control disease in the remaining studies was infection, especially sepsis in two studies (30, 31), which is categorized as ‘hyperferritinemic syndrome,’ similar to MAS or Still’s disease (33). These studies also showed that the serum IL-18 levels in patients with sepsis were approximately 100 pg/mL, which is significantly lower than the levels in AOSD patients. Interestingly, Chen et al. recently reported that serum IL-18 levels at the cut-off value 190.5 pg/ml had high discriminative ability for AOSD and coronavirus disease 2019 (COVID-19), with ROC-AUC 0.948, sensitivity 91.3% and specificity 95.8% (34).

To the best of our knowledge, no comparative study of AOSD, HLH, and MAS has been ever performed in adults, whereas there are numerous studies comparing sJIA, HLH, and MAS in pediatric populations. Krei et al. recently conducted a systematic review of 14 studies regarding the potential role of IL-18 in the diagnosis of HLH or MAS mainly in children (17). In that review, the serum IL-18 levels of the patients with active sJIA were approx. 10^4^–10^5^ pg/mL, whereas those of the HLH patients remained in the range of 10^3^–10^4^ pg/mL. Among the14 studies in the review, Weiss et al. reported that a total IL-18 (i.e., IL-18 bound to IL-18 binding protein [BP]) cut-off value at >11,600 pg/mL distinguished sJIA (or AOSD) or MAS complicating sJIA (or AOSD) from familial or infection-associated HLH, with 88% sensitivity and 93% specificity (35). Considering that AOSD shares pathology with sJIA, these findings seem to support the validity of our present results and further reinforce that sJIA and AOSD are roughly the same disease.

We subdivided the AOSD group and the adult HLH group into four subgroups as shown in **Table 2** and **Figure 1** and observed that the serum IL-18 levels in the AOSD group were significantly higher those in each subdivided adult HLH group, regardless of whether MAS was complicating with AOSD or not. There was no significant difference in serum IL-18 levels between the AOSD MAS- and MAS+ groups. In contrast to these findings, it has been reported that IL-18 or ferritin was significantly increased in AOSD patients with MAS compared to those without MAS (36, 37). These different results might be due to the small sample size of the patients (n=9) and high rate of steroid use at the time of diagnosis (n=3, 33%) in the AOSD MAS+ group.

Regarding useful laboratory data for differentiating AOSD from adult HLH other than IL-18, the present multivariate analysis identified sIL-2R, which is a 45-kDa molecule released from activated T cells (38, 39). SIL-2R is then considered a surrogate biomarker for T cell activation (40). On the other hand, it is also produced by dendritic cells, activated B cells and lymphoma cells (39), and is commonly used as a tumor marker for malignant lymphomas in clinical practice. Although serum sIL-2R levels have been also reported to be elevated not only in malignant lymphomas but also in AOSD or HLH other than LAHS (18, 38), sIL-2R in the LAHS group was markably higher compared to other three groups (espacially the AOSD MAS- group) in this study. Tsuji et al. reported that a sIL-2R/ferritin ratio of patients in LAHS was significantly higher than that of patients in benign disease-associated HLH (8.56 *vs.* 0.66, p=0.0004) (41). Therefore, elevated serum levels of sIL-2R in LAHS might be due in part to its excessive and abnormal secretion from malignant cells.

On the other hand, sIL-2R in the diagnosis of adult HLH has been reported to have high sensitivity but low specificity (42, 43). The same statistical trend was observed in this study (sIL-2R AOSD *vs.* adult HLH; sensitivity: 77.4%, specificity: 69.6%). The results of previous reports and the present study suggest that the ability of sIL-2R to differentiate between AOSD with or without MAS and adult HLH is limited.

Among our patients’ clinical features, the multivariate analysis demonstrated that the presence of arthralgia or arthritis, but not skin rash or sore throat, was an independent differential predictor in the AOSD group. After fever, arthralgia is the second most common symptom in patients with AOSD (44), occurring in more than two-thirds of patients with AOSD and in 35 of the present 46 patients (76.1%). Higher expression levels of IL-18 mRNA were observed in synovial biopsies from patients with active AOSD compared to healthy controls (23), suggesting that IL-18 may be associated with joint pain or arthritis in individuals with AOSD. The combination of these clinical findings with serum IL-18 levels may further improve the diagnostic accuracy for AOSD.

In our present patient population, the serum IL-18 levels in the AOSD group showed a strong correlation not only with ferritin but also with sIL-2R, LDH, and liver deviation enzymes, consistent with previous reports (45), and all of these are considered disease activity markers of AOSD (17, 46). In patients with active AOSD, an increase in serum levels of sIL-2R correlated with IL-18 is presumed to be a mechanism by which IL-18 induces interferon-gamma production by activating T cells with IL-18 receptors, especially IL-17-positive γδ cells (47). In the present study, LDH showed the strongest correlation with IL-18 (r= 0.85, p<0.0001), and the correlation between AST and IL-18 was stronger than that between AST and IL-18. These results might indicate that IL-18 expression is widely distributed not only in the liver but also in organs throughout the body.

The prognosis of AOSD with MAS is worse than that of AOSD without MAS (48), and the mortality rate of AOSD with MAS has been high, ranging from 10% to 41% (49, 50). Therefore, early prediction of progression to MAS is also important in the diagnosis of AOSD (16). IL-18 may also be useful for early diagnosis of AOSD-related MAS, but we could not show any difference in serum IL-18 levels between the AOSD MAS- and AOSD MAS+ group in this study. On the other hand, the HScore is an indicator developed for the diagnosis of secondary HLH, mainly in adults (51), and has recently been reported to be useful for the diagnosis of rheumatic disease-associated HLH, including AOSD (20). Although there were some missing data, the HScore was significantly higher in the AOSD MAS+ group compared to the AOSD MAS- group in the four-group comparison of the current study. The result suggests that the HScore may be useful as a predictor of MAS in the diagnosis of AOSD.

There are several study limitations to address. This was a retrospective and single-center study, and the possibility of unintentional selection bias in the enrollment of patients thus cannot be fully excluded. For example, 17 of the 31 patients (54.8%) in the adult HLH group had LAHS, while Ramos-Casals et al. verified the causative diseases of 2,197 cases of adult HLH or MAS, of which 798 (39.8%) were lymphoma (14). There might have been a partial bias in the underlying diseases of our present adult HLH group. In addition, free IL-18 (i.e., IL-18 not bound to IL-18 BP) in the serum is elevated in patients with AOSD and has been shown to be closely associated with disease activity, and IL-18 BP has been reported to bind to free IL-18 and neutralize its activity (8, 47). The assay that we used in this study could not accurately evaluate the activity of IL-18 because it could not distinguish between free IL-18 not bound to IL-18 BP and IL-18 complexed with IL-18 BP. Finally, eight patients in the AOSD group and eight patients in the adult HLH group were using a steroid or immunosuppressive agent at the time of blood collection. This may have affected the production of cytokines, but as shown in **Supplementary Figure S1**, the ROC-AUC of IL-18 in the untreated AOSD and adult HLH groups with a cutoff value of 18,550 pg/mL was 0.87 (95%CI: 0.78–0.94, 87.0% sensitivity, 92.1% specificity), and there was not a clear difference compared to the cases involving treated patients. There is an opportunity to diagnose AOSD patients and provide treatment interventions, and thus this result might be more in line with daily clinical practice.

## Conclusions

Serum IL-18 levels were shown to be a very useful biomarker for differentiating AOSD from adult HLH. In particular, when MAS is present in AOSD patients, the measurement of serum IL-18 levels may facilitate the distinction between AOSD-associated MAS and other secondary HLH/HPS. Although the diagnosis of AOSD remains challenging, the results of this study may improve the diagnostic performance for AOSD.

## Data Availability Statement

The original contributions presented in the study are included in the study/**Supplementary Material**. Further inquiries can be directed to the corresponding author.

## Ethics Statement

This study complied with the standards of the Declaration of Helsinki, and was registered and approved by the Research Ethics Committee of the Faculty of Medicine of Kindai University (approval no. R02-218). Written informed consent for participation was not required for this study in accordance with the national legislation and the institutional requirements.

## Author Contributions

Conception and design, TS, YN, MF, and IM. Acquisition of data, TS, DT, and KaK. Statistical analysis, TS, YN, and KoK. Manuscript preparation, TS, YN, and MF. All authors contributed to the manuscript’s revision and have read and approved the submitted version

## Conflict of Interest

The authors declare that the research was conducted in the absence of any commercial or financial relationships that could be construed as a potential conflict of interest.

## Publisher’s Note

All claims expressed in this article are solely those of the authors and do not necessarily represent those of their affiliated organizations, or those of the publisher, the editors and the reviewers. Any product that may be evaluated in this article, or claim that may be made by its manufacturer, is not guaranteed or endorsed by the publisher.

## References

[1] Feist E Mitrovic S Fautrel B. Mechanisms, Biomarkers and Targets for Adult-Onset Still’s Disease. Nat Rev Rheumatol (2018) 14(10):603–18. doi: 10.1038/s41584-018-0081-x PMC7097309 30218025

[2] Giacomelli R Ruscitti P Shoenfeld Y. A Comprehensive Review on Adult Onset Still’s Disease. J Autoimmun (2018) 93:24–36. doi: 10.1016/j.jaut.2018.07.018 30077425

[3] Gerfaud-Valentin M Jamilloux Y Iwaz J Sève P. Adult-Onset Still’s Disease. Autoimmun Rev (2014) 13(7):708–22. doi: 10.1016/j.autrev.2014.01.058 24657513

[4] Hu Q Shi H Zeng T Liu H Su Y Cheng X. Increased Neutrophil Extracellular Traps Activate NLRP3 and Inflammatory Macrophages in Adult-Onset Still’s Disease. Arthritis Res Ther (2019) 21(1):9. doi: 10.1186/s13075-018-1800-z 30616678 PMC6323819

[5] Yamaguchi M Ohta A Tsunematsu T Kasukawa R Mizushima Y Kashiwagi H. Preliminary Criteria for Classification of Adult Still’s Disease. J Rheumatol (1992) 19(3):424–30. 1578458

[6] Fautrel B Zing E Golmard JL Le Moel G Bissery A Rioux C. Proposal for a New Set of Classification Criteria for Adult-Onset Still Disease. Med (Baltimore) (2002) 81(3):194–200. doi: 10.1097/00005792-200205000-00003 11997716

[7] Fautrel B. Ferritin Levels in Adult Still’s Disease: Any Sugar? Joint Bone Spine (2002) 69(4):355–7. doi: 10.1016/s1297-319x(02)00409-8 12184429

[8] Girard C Rech J Brown M Allali D Roux-Lombard P Spertini F. Elevated Serum Levels of Free Interleukin-18 in Adult-Onset Still’s Disease. Rheumatol (Oxford) (2016) 55(12):2237–47. doi: 10.1093/rheumatology/kew300 27616144

[9] Maria AT Le Quellec A Jorgensen C Touitou I Rivière S Guilpain P. Adult Onset Still’s Disease (AOSD) in the Era of Biologic Therapies: Dichotomous View for Cytokine and Clinical Expressions. Autoimmun Rev (2014) 13(11):1149–59. doi: 10.1016/j.autrev.2014.08.032 25183244

[10] Colafrancesco S Priori R Alessandri C Perricone C Pendolino M Picarelli G. IL-18 Serum Level in Adult Onset Still’s Disease: A Marker of Disease Activity. Int J Inflamm (2012) 2012:156890. doi: 10.1155/2012/156890 PMC3385601 22762008

[11] Nam SW Kang S Lee JH Yoo DH. Different Features of Interleukin-37 and Interleukin-18 as Disease Activity Markers of Adult-Onset Still’s Disease. J Clin Med (2021) 10(5):910. doi: 10.3390/jcm10050910 33652679 PMC7956170

[12] Jung KH Kim JJ Lee JS Park W Kim TH Jun JB. Interleukin-18 as an Efficient Marker for Remission and Follow-Up in Patients With Inactive Adult-Onset Still’s Disease. Scand J Rheumatol (2014) 43(2):162–9. doi: 10.3109/03009742.2013.824023 24134323

[13] Gabay C Fautrel B Rech J Spertini F Feist E Kötter I. Open-Label, Multicentre, Dose-Escalating Phase II Clinical Trial on the Safety and Efficacy of Tadekinig Alfa (IL-18BP) in Adult-Onset Still’s Disease. Ann Rheumatic Dis (2018) 77:840–7. doi: 10.1136/annrheumdis-2017-212608 PMC5965361 29472362

[14] Ramos-Casals M Brito-Zerón P López-Guillermo A Khamashta MA Bosch X. Adult Haemophagocytic Syndrome. Lancet (2014) 383(9927):1503–16. doi: 10.1016/s0140-6736(13)61048-x 24290661

[15] La Rosee P Horne A Hines M von Bahr Greenwood T Machowicz R Berliner N. Recommendations for the Management of Hemophagocytic Lymphohistiocytosis in Adults. Blood (2019) 133(23):2465–77. doi: 10.1182/blood.2018894618 30992265

[16] Crayne CB Albeituni S Nichols KE Cron RQ. The Immunology of Macrophage Activation Syndrome. Front Immunol (2019) 10:119. doi: 10.3389/fimmu.2019.00119 30774631 PMC6367262

[17] Krei JM Moller HJ Larsen JB. The Role of Interleukin-18 in the Diagnosis and Monitoring of Hemophagocytic Lymphohistiocytosis/Macrophage Activation Syndrome - a Systematic Review. Clin Exp Immunol (2021) 203(2):174–82. doi: 10.1111/cei.13543 PMC7806447 33128796

[18] Henter JI Horne A Aricó M Egeler RM Filipovich AH Imashuku S. HLH-2004: Diagnostic and Therapeutic Guidelines for Hemophagocytic Lymphohistiocytosis. Pediatr Blood Cancer (2007) 48(2):124–31. doi: 10.1002/pbc.21039 16937360

[19] Rau M Schiller M Krienke S Heyder P Lorenz H Blank N. Clinical Manifestations But Not Cytokine Profiles Differentiate Adult-Onset Still’s Disease and Sepsis. J Rheumatol (2010) 37(11):2369–76. doi: 10.3899/jrheum.100247 20810496

[20] Batu ED Erden A Seyhoğlu E Kilic L Büyükasık Y Karadag O. Assessment of the HScore for Reactive Haemophagocytic Syndrome in Patients With Rheumatic Diseases. Scand J Rheumatol (2017) 46(1):44–8. doi: 10.3109/03009742.2016.1167951 27359073

[21] Mazodier K Marin V Novick D Farnarier C Robitail S Schleinitz N. Severe Imbalance of IL-18/IL-18BP in Patients With Secondary Hemophagocytic Syndrome. Blood (2005) 106(10):3483–9. doi: 10.1182/blood-2005-05-1980 PMC1895045 16020503

[22] Kawashima M Yamamura M Taniai M Yamauchi H Tanimoto T Kurimoto M. Levels of Interleukin-18 and its Binding Inhibitors in the Blood Circulation of Patients With Adult-Onset Still’s Disease. Arthritis Rheum (2001) 44(3):550–60. doi: 10.1002/1529-0131(200103)44:3<550::Aid-anr103>3.0.Co;2-5 11263769

[23] Chen DY Lan JL Lin FJ Hsieh TY. Proinflammatory Cytokine Profiles in Sera and Pathological Tissues of Patients With Active Untreated Adult Onset Still’s Disease. J Rheumatol (2004) 31(11):2189–98. 15517632

[24] Maruyama J Inokuma S. Cytokine Profiles of Macrophage Activation Syndrome Associated With Rheumatic Diseases. J Rheumatol (2010) 37(5):967–73. doi: 10.3899/jrheum.090662 20231207

[25] Chen DY Lin CC Chen YM Lan JL Hung WT Chen HH. Involvement of TLR7 MyD88-Dependent Signaling Pathway in the Pathogenesis of Adult-Onset Still’s Disease. Arthritis Res Ther (2013) 15(2):R39. doi: 10.1186/ar4193 23497717 PMC3672755

[26] Saiki O Uda H Nishimoto N Miwa T Mima T Ogawara T. Adult Still’s Disease Reflects a Th2 Rather Than a Th1 Cytokine Profile. Clin Immunol (2004) 112(1):120–5. doi: 10.1016/j.clim.2004.03.023 15207789

[27] Chen DY Lan JL Lin FJ Hsieh TY. Association of Intercellular Adhesion Molecule-1 With Clinical Manifestations and Interleukin-18 in Patients With Active, Untreated Adult-Onset Still’s Disease. Arthritis Rheum (2005) 53(3):320–7. doi: 10.1002/art.21164 15934126

[28] Conigliaro P Priori R Bombardieri M Alessandri C Barone F Pitzalis C. Lymph Node IL-18 Expression in Adult-Onset Still’s Disease. Ann Rheum Dis (2009) 68(3):442–3. doi: 10.1136/ard.2008.093781 19213748

[29] Kim HA An JM Nam JY Jeon JY Suh CH. Serum S100A8/A9, But Not Follistatin-Like Protein 1 and Interleukin 18, may be a Useful Biomarker of Disease Activity in Adult-Onset Still’s Disease. J Rheumatol (2012) 39(7):1399–406. doi: 10.3899/jrheum.120079 22660800

[30] Priori R Colafrancesco S Alessandri C Minniti A Perricone C Iaiani G. Interleukin 18: A Biomarker for Differential Diagnosis Between Adult-Onset Still’s Disease and Sepsis. J Rheumatol (2014) 41(6):1118–23. doi: 10.3899/jrheum.130575 24786926

[31] Koga T Sumiyoshi R Furukawa K Sato S Migita K Shimizu T. Interleukin-18 and Fibroblast Growth Factor 2 in Combination Is a Useful Diagnostic Biomarker to Distinguish Adult-Onset Still’s Disease From Sepsis. Arthritis Res Ther (2020) 22(1):108. doi: 10.1186/s13075-020-02200-4 32381117 PMC7206754

[32] Chen PK Lan JL Li JP Chang CK Chang SH Huang PH. Elevated Plasma Galectin-3 Levels and Their Correlation With Disease Activity in Adult-Onset Still’s Disease. Clin Rheumatol (2020) 39(6):1945–52. doi: 10.1007/s10067-020-04946-3 31960208

[33] Rosário C Zandman-Goddard G Meyron-Holtz EG D’Cruz DP Shoenfeld Y. The Hyperferritinemic Syndrome: Macrophage Activation Syndrome, Still’s Disease, Septic Shock and Catastrophic Antiphospholipid Syndrome. BMC Med (2013) 11:185. doi: 10.1186/1741-7015-11-185 23968282 PMC3751883

[34] Chen PK Lan J-L Huang P-H Hsu J-L Chang C-K Tien N. Interleukin-18 Is a Potential Biomarker to Discriminate Active Adult-Onset Still’s Disease From COVID-19. Front Immunol (2021) 12:719544. doi: 10.3389/fimmu.2021.719544 34367188 PMC8343229

[35] Weiss ES Girard-Guyonvarc’h C Holzinger D de Jesus AA Tariq Z Picarsic J. Interleukin-18 Diagnostically Distinguishes and Pathogenically Promotes Human and Murine Macrophage Activation Syndrome. Blood (2018) 131(13):1442–55. doi: 10.1182/blood-2017-12-820852 PMC5877443 29326099

[36] Shimizu M Nakagishi Y Inoue N Mizuta M Ko G Saikawa Y. Interleukin-18 for Predicting the Development of Macrophage Activation Syndrome in Systemic Juvenile Idiopathic Arthritis. Clin Immunol (2015) 160(2):277–81. doi: 10.1016/j.clim.2015.06.005 26101092

[37] Colafrancesco S Priori R Alessandri C Astorri E Perricone C Blank M. Scd163 in AOSD: A Biomarker for Macrophage Activation Related to Hyperferritinemia. Immunol Res (2014) 60(2-3):177–83. doi: 10.1007/s12026-014-8563-7 25388964

[38] Choi JH Suh CH Lee YM Suh YJ Lee SK Kim SS. Serum Cytokine Profiles in Patients With Adult Onset Still’s Disease. J Rheumatol (2003) 30(11):2422–7. 14677188

[39] Lin M Park S Hayden A Giustini D Trinkaus M Pudek M. Clinical Utility of Soluble Interleukin-2 Receptor in Hemophagocytic Syndromes: A Systematic Scoping Review. Ann Hematol (2017) 96(8):1241–51. doi: 10.1007/s00277-017-2993-y 28497365

[40] Coca A Bundy KW Marston B Huggins J Looney RJ. Macrophage Activation Syndrome: Serological Markers and Treatment With Anti-Thymocyte Globulin. Clin Immunol (2009) 132(1):10–8. doi: 10.1016/j.clim.2009.02.005 19297252

[41] Tsuji T Hirano T Yamasaki H Tsuji M Tsuda H. A High sIL-2R/Ferritin Ratio is a Useful Marker for the Diagnosis of Lymphoma-Associated Hemophagocytic Syndrome. Ann Hematol (2014) 93(5):821–6. doi: 10.1007/s00277-013-1925-8 PMC3976506 24705932

[42] Hayden A Lin M Park S Pudek M Schneider M Jordan MB. Soluble Interleukin-2 Receptor is a Sensitive Diagnostic Test in Adult HLH. Blood Adv (2017) 1(26):2529–34. doi: 10.1182/bloodadvances.2017012310 PMC5728644 29296904

[43] Naymagon L Tremblay D Troy K Mascarenhas J. Soluble Interleukin-2 Receptor (sIL-2r) Level Is a Limited Test for the Diagnosis of Adult Secondary Hemophagocytic Lymphohistiocytosis. Eur J Haematol (2020) 105(3):255–61. doi: 10.1111/ejh.13433 32353917

[44] Fautrel B. Adult-Onset Still Disease. Best Pract Res Clin Rheumatol (2008) 22(5):773–92. doi: 10.1016/j.berh.2008.08.006 19028363

[45] Shimizu M Yokoyama T Yamada K Kaneda H Wada H Wada T. Distinct Cytokine Profiles of Systemic-Onset Juvenile Idiopathic Arthritis-Associated Macrophage Activation Syndrome With Particular Emphasis on the Role of Interleukin-18 in its Pathogenesis. Rheumatol (Oxford) (2010) 49(9):1645–53. doi: 10.1093/rheumatology/keq133 20472718

[46] Kasama T Furuya H Yanai R Ohtsuka K Takahashi R Yajima N. Correlation of Serum CX3CL1 Level With Disease Activity in Adult-Onset Still’s Disease and Significant Involvement in Hemophagocytic Syndrome. Clin Rheumatol (2012) 31(5):853–60. doi: 10.1007/s10067-012-1952-1 22322207

[47] Kaplanski G. Interleukin-18: Biological Properties and Role in Disease Pathogenesis. Immunol Rev (2018) 281(1):138–53. doi: 10.1111/imr.12616 PMC7165732 29247988

[48] Dhote R Simon J Papo T Detournay B Sailler L Andre MH. Reactive Hemophagocytic Syndrome in Adult Systemic Disease: Report of Twenty-Six Cases and Literature Review. Arthritis Rheum (2003) 49(5):633–9. doi: 10.1002/art.11368 14558048

[49] Ruscitti P Rago C Breda L Cipriani P Liakouli V Berardicurti O. Macrophage Activation Syndrome in Still’s Disease: Analysis of Clinical Characteristics and Survival in Paediatric and Adult Patients. Clin Rheumatol (2017) 36(12):2839–45. doi: 10.1007/s10067-017-3830-3 28914368

[50] Ruscitti P Cipriani P Ciccia F Masedu F Liakouli V Carubbi F. Prognostic Factors of Macrophage Activation Syndrome, at the Time of Diagnosis, in Adult Patients Affected by Autoimmune Disease: Analysis of 41 Cases Collected in 2 Rheumatologic Centers. Autoimmun Rev (2017) 16(1):16–21. doi: 10.1016/j.autrev.2016.09.016 27664384

[51] Fardet L Galicier L Lambotte O Marzac C Aumont C Chahwan D. Development and Validation of the HScore, a Score for the Diagnosis of Reactive Hemophagocytic Syndrome. Arthritis Rheumatol (2014) 66(9):2613–20. doi: 10.1002/art.38690 24782338

