# Supplementary material for: Usefulness of Interleukin-18 as a Diagnostic Biomarker to Differentiate Adult-Onset Still’s Disease With/Without Macrophage Activation Syndrome From Other Secondary Hemophagocytic Lymphohistiocytosis in Adults
Source: Front Immunol. 2021 Oct 8;12:750114. doi: 10.3389/fimmu.2021.750114 (PMC8533049; doi:10.3389/fimmu.2021.750114)
Supplement: Supplementary file 1 [file DataSheet_1.docx]

Supplementary Material

- 1. **Supplementary Tables**

**Supplementary TABLE S1 |** The details of 9 patients in the AOSD MAS+ group who met at least 5 out of 7 the HLH 2004 criteria (excluding low or absent NK-cell activity).

| Case | Fever≧38.5℃ | Spleno-megaly | Cytopenias at least 2 of 3 lineages  Hb<9g/dL  PLT<100,000/µL  ANC<1,000/µL | Ferritin  >500 ng/mL | Trig>265mg/dL  and/ or  Fib<150mg/dL | Hemophago-cytosis  in  bone marrow | sIL-2R  >2,400  U/mL | Total |
| --- | --- | --- | --- | --- | --- | --- | --- | --- |
|  | **+** | **+** | (Hb; 14.0)  (PLT; 119,000)  (ANC; 3620) | **+; 55,963** | (Trig; 166)  **Fib; 138** | **+** | **+; 35,545** | **6/7** |
|  | **+** | - | **Hb; 8.5**  **PLT; 30,000**  (ANC; 1,045) | **+; 7,348** | (Trig; 131)  (Fib; 237) | **+** | **+; 4,440** | **5/7** |
|  | **+** | **+** | (Hb; 11.8)  (PLT; 243,000)  (ANC; 33,748)) | **+; 52,516** | (Trig;241)  (Fib; 668) | **+** | **+; 2,442** | **5/7** |
|  | **+** | **+** | **Hb; 8.0**  **PLT; 79,000**  (ANC; 12,642) | **+: 119,267** | (Trig; 209)  (Fib; 274) | **+** | **+; 5,233** | **6/7** |
|  | **+** | - | **Hb; 7.7**  **PLT; 51,000**  (ANC; 3,548) | **+; 20,613** | (Trig; 110)  **Fib:146** | **+** | -; 810 | **5/7** |
|  | **+** | **+** | **Hb; 7.5**  **PLT; 97,000**  (ANC; 4,025) | **+; 35,998** | (Trig; 149)  (Fib; 264) | **+** | -; 1,708 | **5/7** |
|  | **+** | - | **Hb; 8.6**  **PLT; 59,000**  (ANC; 31,398) | **+; 35,328** | (Trig; 207)  (Fib; 385) | **+** | **+; 6,635** | **5/7** |
|  | **+** | **+** | (Hb; 10.7)  (PLT; 180,000)  (ANC; 6,034) | **+; 13,791** | *(Trig; data missing)*  **Fib; 148** | **+** | -; 2,103 | **5/7** |
|  | **+** | - | **Hb; 8.8**  **PLT; 76,000**  (ANC; 9,306) | **+; 5,000** | (Trig; 212)  (Fib; 186) | **+** | **+; 5,310** | **5/7** |

+; means that the endpoint was met, - or (); means that the endpoint was not met. ANC: absolute neutrophil count, Fib; fibrinogen, Hb; hemoglobin, PLT; platelet, Trig; triglyceride.

**Supplementary TABLE S2 ｜** Underlying diseases and serum IL-18, sIL-2R, and ferritin levels in the non-LAHS group patients

| **Underlying diseases** | **IL-18, pg/ml** | **sIL-2R, U/mL** | **Ferritin, ng/mL** |
| --- | --- | --- | --- |
| **Rheumatic diseases** | | | |
| SLE | 2,970 | 1,540 | 10,033 |
| , SLE | 2,370 | 4,397 | 3,711 |
| SLE | 1,380 | 2,157 | 7,365 |
| SLE | 1,040 | 1,633 | 7,837 |
| SLE | 985 | 1,633 | 2,245 |
| Dermatomyositis | 510 | 1,726 | 1,765 |
| **Infections** | | | |
| Multiple muscle abscesses | 64,856 | 5,561 | 7,026 |
| Sepsis | 1,830 | 6,602 | 3,701 |
| Sepsis | 436 | 1,368 | 2,666 |
| **Others** | | | |
| Breast cancer (metastasis) | 206,000 | 2,633 | 33,901 |
| After HSCT | 196 | 966 | 1,316 |
| Unknown | 294,000 | 3,762 | 21,034 |
| Unknown | 1,860 | 6,383 | 1,668 |
| Unknown | 1,650 | 19,990 | 483 |

IL: interleukin, sIL-2R: soluble interleukin 2 receptor, SLE: systemic lupus erythematosus, HSCT: hematopoietic stem cell transplantation.

## 1.2 Supplementary Figure


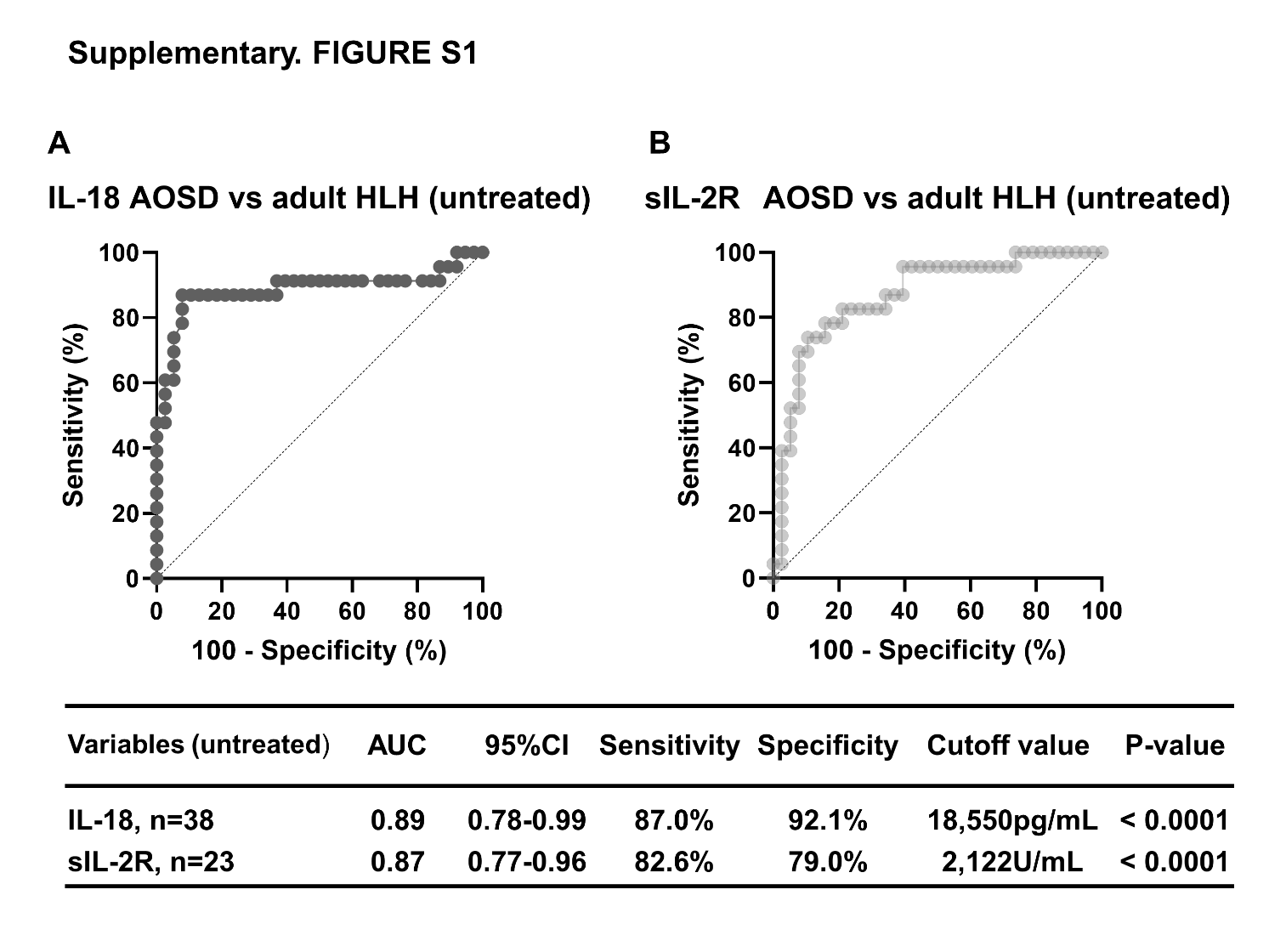


**Supplementary Figure S1 |** The ROC curve analysis for the differential diagnosis of the AOSD and adult HLH groups (untreated) based on the serum levels of IL-18 and sIL-2R. The predictive performance of the serum levels of **(A)** IL-18 and **(B)** sIL-2R was validated by the ROC analysis, and the accuracy of differential diagnosis is indicated by the AUC and 95%CI.
